# Supplementary material for: Rapid modulation of choice behavior by ultrasound on the human frontal eye fields
Source: Nat Commun. 2026 Feb 20;17:2966. doi: 10.1038/s41467-026-69854-7 (PMC13035901; doi:10.1038/s41467-026-69854-7)
Supplement: Supplementary file 1 — Supplementary Information [file 41467_2026_69854_MOESM1_ESM.pdf]

Supplementary Information

Manuscript: *Rapid modulation of choice behavior by ultrasound on the human frontal eye fields*

Supplementary Sections

- **S.1 Zero-delay trials** p.2
- **S.2 Estimating the bias, slope and lapse rate** p.3
- **S.3 Ipsilateral after-effects and stimulation perception** p.4
- **S.4 Model diagnostics** p.5

Supplementary Figures

- **Figure S1.** Empirical psychometric curves of FEF and control-site (M1) stimulation. p.6
- **Figure S2.** Differences in TUS effects for zero-delay and non-zero-delay trials. p.7
- **Figure S3.** Estimating bias, slope and lapse rate following stimulation. p.8
- **Figure S4.** Participant accuracy and psychometric quality checks. p.10
- **Figure S5.** Schematic overview of session duration and block structure. p.12
- **Figure S6.** Schematic overview of auditory masking structure in TUS and sham trials. p.13
- **Figure S7.** Schematic overview of functional localizers and MRI protocol. p.14
- **Figure S8.** Overview of the study setup. p.15
- **Figure S9.** Raw saccade examples and spatial endpoint distributions. p.16
- **Figure S10.** GLMM diagnostics for the main statistical models. p.17
- **Figure S11.** MRS-derived measurements for left FEF and left M1 (including QC/summary). p.18
- **Figure S12.** Transducer/drive-system specifications and stimulation parameters. p.19

Supplementary Tables

- **Table S1.** Additional analyses and statistical outcomes examining FEF stimulation effects. p.20
- **Table S2.** As Table S1, excluding trials where target delay was 0 s. p.20
- **Table S3.** Additional analyses comparing FEF vs M1 stimulation. p.20
- **Table S4.** As Table S3, excluding trials where target delay was 0 s. p.21
- **Table S5.** Additional analyses on FEF vs M1 stimulation including additional covariates. p.21
- **Table S6.** Interaction between stimulation condition and baseline FEF GABA+ on choice behavior p.22
- **Table S7.** As Table S6, excluding trials where target delay was 0 s. p.22
- **Table S8.** Interaction between stimulation condition and baseline M1 GABA+ on choice behavior. p.22
- **Table S9.** Interaction between stimulation condition (M1 vs sham) and baseline M1 GABA+ on choice behavior (excluding target-delay 0 s). p.23

S.1 Zero-delay trials

Our analyses are tailored to the choice domain, where the SOA provides informative—but not overwhelming—evidence in favor of one saccade direction over the other. When there is no meaningful choice to be made, i.e., when the SOA is long, performance approaches ceiling, and we did not observe any modulatory effect of TUS (Supplementary Information S.2).

In contrast, exploratory analyses revealed that when no correct choice can be made (i.e., when the two cues are presented simultaneously), TUS over both FEF and M1 biased saccades. A three-way interaction trend between region (FEF/M1), stimulation side (left/right), and delay (non-zero/zero) suggested that any M1 effect was limited to the zero-delay trials (b = -0.50, 95%-CI [-1.02, 0.02], $\chi^2(1)$ = 3.6, p = 0.058; Fig. S2). However, this analysis is underpowered due to the inclusion of multiple interactions. To address this, we split the data into FEF and M1 subsets. These subsequent tests confirmed that M1 TUS effects are specific to zero-delay trials (condition (left M1/right M1) x delay (non-zero/zero): b = -0.57, 95%-CI [-0.94, -0.20], $\chi^2(1)$ = 9.0, p = 0.002; Fig. S2). In contrast, FEF TUS effects persisted across both zero- and non-zero delay trials (condition (left FEF/right FEF) x delay (non-zero/zero): b = -0.08, 95%-CI [-0.45, 0.28], $\chi^2(1)$ = 0.2, p = 0.6; Fig. S2), indicating that the observed effects of FEF TUS are robust and stable.

The M1 TUS effect on zero-delay trials was significant, both in statistical and absolute terms. When these trials are included in the choice domain, the shared direction of TUS bias across FEF and M1 obscures a putative interaction of stimulation side and region (side (left/right) x region (FEF/M1): b = 0.16, 95%-CI [-0.03, 0.36], $\chi^2(1)$ = 2.9, p = 0.09). Instead, it reveals a main effect of stimulation side (side (left/right): b = -0.26, 95%-CI [-0.41, -0.11], $\chi^2(1)$ = 11.5, p = 0.007), and region (region (FEF/M1): b = -0.14, 95%-CI [-0.26, -0.01], $\chi^2(1)$ = 4.6, p = 0.032).

The highly specific biasing effect of TUS over M1, observed only when the two visual cues are presented simultaneously, warrants further investigation. In these conditions, no correct choice can be made based on the visual cues alone. One possible explanation is that an M1 TUS bias arises from true neuromodulation of M1. Indeed, M1 circuits anatomically converge with downstream saccade circuits in the basal ganglia to support eye-hand coordination[101]. Perhaps when visual information is absent, motor biases are propagated through these shared effector circuits.

S.2 Estimating the bias, slope and lapse rate

To ascertain the specificity of the TUS effects on biasing saccade direction, we explored whether FEF TUS influences choice bias (a horizontal shift in the decision curve), target discrimination (a change in slope), or bias beyond the choice domain (a change in asymptotes/lapse).

Bias and slope were analyzed using mixed effects logistic regression focusing on trials with short delays (-75 to +75 ms). Choice bias was assessed by examining the main effect of stimulation condition ($\chi^2(1) = 22.2$, $p < 0.001$), revealing that TUS induced a horizontal shift in the decision curve. Specifically, left FEF stimulation shifted the curve by -3.67 ms, whereas right FEF stimulation shifted it by +3.77 ms. Post hoc tests showed that the shift between left and right FEF stimulation was significantly different ($p < 0.001$; Fig. S3A), unlike the shift between left and right M1 stimulation ($p = 0.09$; Fig. S3A).

In contrast, the slope of the decision curves, reflecting target discrimination was unaffected by TUS, as indicated by the non-significant interaction between stimulation condition and target delay (condition x SOA: $\chi^2(1) = 2.715$, $p = 0.6$; condition (left FEF) x SOA: $b = 0.001$, 95%-CI [-0.002, 0.003]; condition (right FEF) x SOA: $b = 0.001$, 95%-CI [-0.002, 0.004]; condition (left M1) x SOA: $b = 0.001$, 95%-CI [-0.002, 0.003]; condition (right M1) x SOA: $b = 0.002$, 95%-CI [-0.001, 0.005]; Fig. S3B).

Finally, to determine if TUS alters bias outside the choice domain, we analyzed trials with longer absolute delays (75 to 200 ms). While there was a trend suggesting a condition effect (condition: $\chi^2(1) = 9.0$, $p = 0.060$; condition (left FEF): $b = 0.23$, 95%-CI [-0.01, 0.0.56]; condition (right FEF): $b = 0.34$, 95%-CI [0.03, 0.64]; condition (left M1): $b = 0.33$, 95%-CI [0.02, 0.64]; condition (right M1): $b = 0.35$, 95%-CI [0.04, 0.65]; Fig. S3C), post hoc tests confirmed no significant impact of TUS on choice bias these trials (FEF (left/right): $p = 1.0$; M1(left/right): $p = 1.0$; Fig. S3C). This reinforces the conclusion that TUS selectively biases responses under conditions of response uncertainty and is unlikely to reverse or evoke responses under high certainty.

S.3 Ipsilateral after-effects and stimulation perception

Within each block, participants received pseudorandomized left TUS, right TUS, and sham stimulation. To investigate potential longer-lasting TUS effects beyond the stimulation duration itself, we analyzed sham trials that directly followed a TUS trial. First, as a baseline we tested the sham→sham condition, and found no significant bias following sham trials a non-significant intercept on the logit scale (b = −0.07, p = 0.3), corresponding to p = 0.5 (95%-CI [0.455–0.512]) from 0.5.

Interestingly, for sham trials following TUS, we observed a significant increase in ipsilateral responses during these sham trials—for example, if a sham trial followed a left TUS trial, participants were more likely to make a leftward saccade ($side_{t-1}$: b = -0.10, 95%-CI [-0.18, -0.02], $\chi^2(1)$ = 5.3, p = 0.021; Fig. 6A). This ipsilateral bias on sham trials following TUS was in the opposite direction of the behavioral effects induced by FEF TUS, which increased contralateral saccades. One could conceive that this reversal reflects compensatory carry-over effects of TUS. Importantly, however, these after-effects and stimulation perception biases did not differ between FEF and M1 conditions ($region_{t-1}$: b = 0.8, 95%-CI [-0.08, 0.11], $\chi^2(1)$ = 0.1, p = 0.8; $side_{t-1}$ x $region_{t-1}$: b = 0.02, 95%-CI [-0.06, 0.10], $\chi^2(1)$ = 0.3, p = 0.6), whereas TUS effects were present only for FEF stimulation. Thus, the robust FEF TUS effects cannot be explained by these ipsilateral tendencies, nor do they support the presence of post-TUS compensatory mechanisms.

Thus, while neuromodulatory effects of TUS cannot explain the presence of ipsilateral after-effects, this begs the question what does drive these effects. We speculate that the answer to this question in the similar ipsilateral response pattern that emerged in the masking assessment. Here, participants performed a forced-choice task to report whether they perceived the stimulation as originating from left or right TUS. Participants significantly misattributed stimulation to the ipsilateral side, independent of stimulation region (side: b = -1.2, 95%-CI [-2.1, -0.3], $\chi^2(1)$ = 7.0, p = 0.008; side (left/right) x region (FEF/M1): b = -0.5, 95%-CI [-1.5, 0.5], $\chi^2(1)$ = 1.1, p = 0.3; Fig. 6C).

This ipsilateral and lateralized perception of TUS likely stems from specific properties of skull morphology. Variations in how flexural waves—vibrations traveling through the skull—are transmitted can cause the highest amplitude near the contralateral cochlea, influencing perceived sound location[39]. We speculate that the regionally non-specific but lateralized after-effects observed in sham trials immediately post TUS may reflect an attention-orienting response. If participants subjectively perceived prior stimulation as originating from the left, they may have been biased toward making leftward saccades afterward.

S.4 Model diagnostics

For binomial generalized linear mixed models we used simulation-based residual diagnostics (DHARMa) to verify model assumptions. We assessed overall model fits by inspecting the uniformity of simulated residuals (DHARMa QQ/uniformity plot with a Kolmogorov–Smirnov test), and tested for (over/under)dispersion and outliers using DHARMa's non-parametric procedures. To evaluate homoscedasticity and potential model mis-specification, we plotted residuals versus fitted values (rank-transformed). Linearity of continuous predictors (e.g., SOA_scaled) was examined both visually via residuals versus predictor plots and formally by comparing the linear specification with a natural-spline alternative (likelihood-ratio test). We also inspected residual distributions across factor levels (e.g., stimulation side) using within-group uniformity checks and a Levene test for equality of variances. Finally, we evaluated multicollinearity of fixed effects via variance inflation factors (VIFs). All diagnostics were implemented in R with the packages DHARMa, performance/see, and influence.ME. Together, these diagnostics indicated adequate fit for both the left vs. right FEF model, and the Side × Region model. Residuals are approximately uniform with no over/under-dispersion or outlier excess, no systematic trend versus fitted values or SOA, and no heteroscedasticity. See Fig. S10 for detailed results.

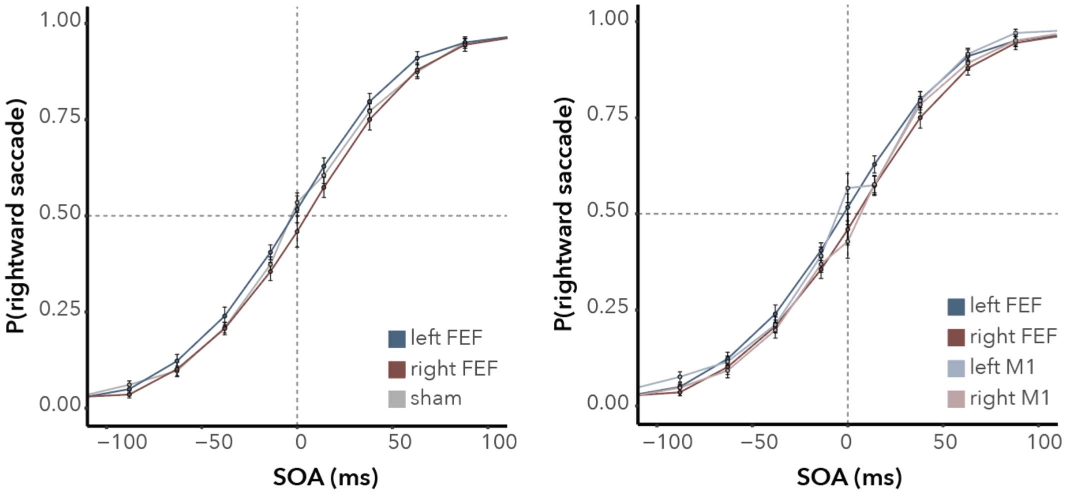

Figure S1 | Empirical psychometric curves of FEF and control conditions
Left panel shows FEF vs sham TUS effects, right panel shows FEF vs M1 TUS effects. Data are binned for visualization purposes into bins of approximately equal numbers of trials, resulting in SOA intervals of [0], [8.3 – 25.0], [33.3 – 50.0], [58.3 – 75.0], [83.3 – 100.0], [108.3 – 141.7], and [150 – 200]. Bins are symmetric for negative values. Note that all inferential statistics were performed using continuous SOAs. Dots represent the group mean per bin, and error bars indicate the S.E.M. across participants (n = 35). Source data are provided as a Source Data file.

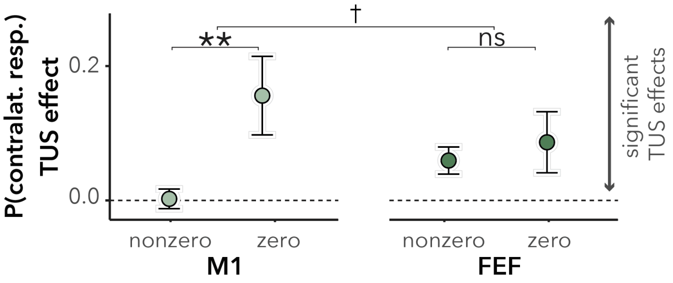

Figure S2 | Differences in TUS effects for zero-delay and nonzero-delay trials in FEF and M1

TUS effects are expressed as the probability of making contralateral saccades (e.g., left hemisphere is stimulated and a rightward saccade is made; *n* = 35). A contralateral saccade probability greater than 0.5 indicates a TUS effect beyond chance. Left and right FEF and M1 conditions were pooled to form FEF and M1 categories, respectively. For M1, a significant difference in TUS effects was observed between zero-delay and non-zero-delay trials, with TUS effects being present only for zero-delay trials, indicating a delay-dependent modulation of M1. In contrast, FEF stimulation produced significant TUS effects for both zero-delay and non-zero-delay trials, with no significant difference between the delays, suggesting a stable and consistent modulation of FEF regardless of delay. Data are presented as group means with standard error of the mean (S.E.M.). ** $p<0.01$, † $p<0.1$, ns $p>0.1$. Source data are provided as a Source Data file. Exact *p* values are presented in the supplementary text. Statistical significance was determined using two-sided logistic mixed effects regressions. No multiple comparisons were applied.

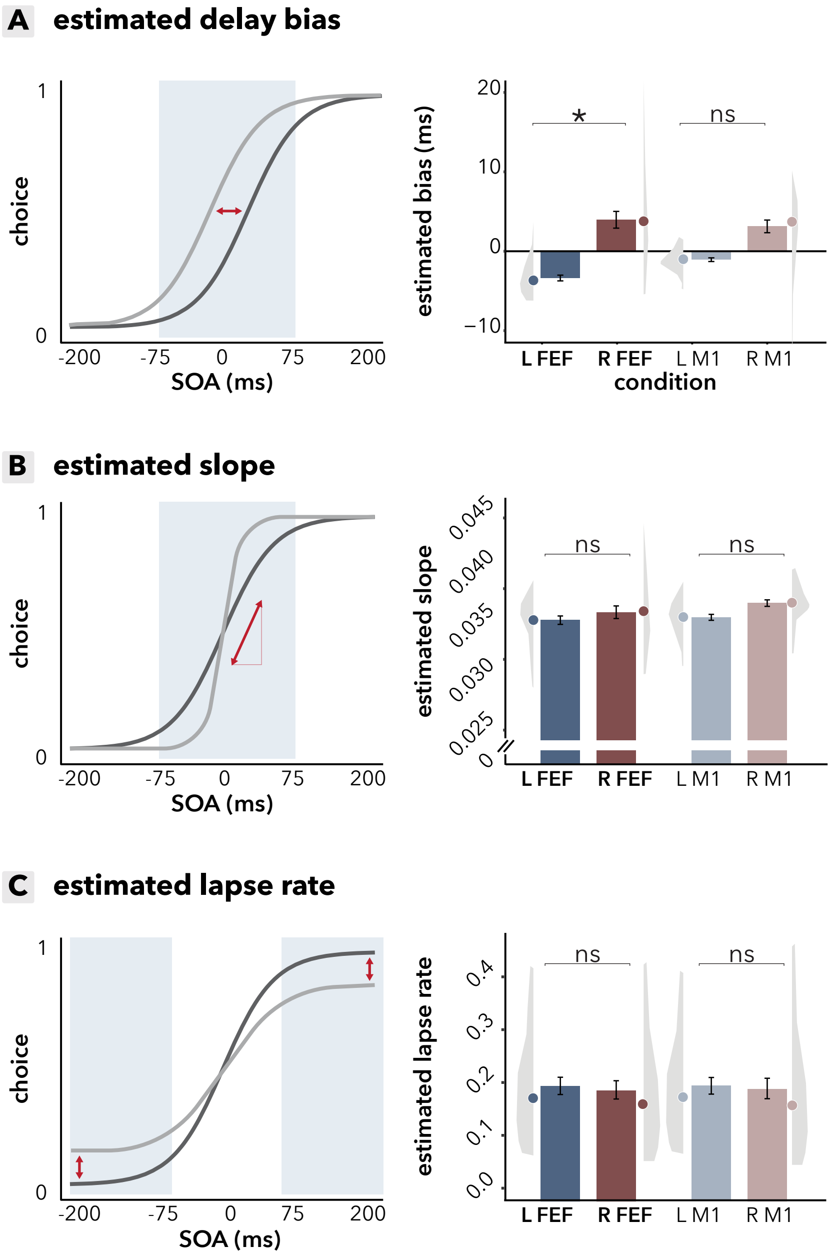

Figure S3 | Estimating bias, slope and lapse rate following FEF and M1 TUS

(A) Choice bias (n = 35). Left: a visual representation of a horizontal shift in the decision curve, with the shaded blue area indicating the region of interest for the analysis. Right: estimated bias per participant derived from the mixed-effects model, which includes random effects (individual distributions with cloud and SEM) and fixed effects (group mean represented by the dot). Left FEF stimulation shifted the decision curve by -3.67 ms, while right FEF stimulation shifted it by +3.77 ms. A significant difference was found between left and right FEF ($p < 0.001$), but no significant shift was observed for M1 ($p = 0.09$), suggesting that TUS specifically affects choice bias in FEF. **(B)** Target discrimination (slope; n = 35). Left: a visual representation of how changes in slope would look, with the blue shaded area marking the region of interest. Right: estimated slopes per participant derived from the mixed-effects model, which includes random effects (individual distributions with cloud and SEM) and fixed effects (group mean represented by the dot). No significant effect of TUS on slope was observed, as no difference was found between these conditions ($p = 0.6$), indicating that TUS does not significantly impact target discrimination. **(C)** Bias beyond the choice domain (lapse rate; n = 35). Left: a visual representation of changes in lapse rate, with the shaded blue area marking the region of interest. Right: estimated lapse rates per participant derived from the mixed-effects model, which includes

random effects (individual distributions with cloud and SEM) and fixed effects (group mean represented by the dot). While a trend toward a condition effect was observed (p = 0.060), post hoc tests confirmed no significant impact of TUS on lapse rate (p = 1.0 for both FEF and M1), reinforcing the conclusion that TUS selectively biases choice behavior without affecting performance in the lapse rate domain. Source data are provided as a Source Data file. Exact p values are presented in the supplementary text. Statistical significance was determined using two-sided logistic mixed effects regressions. No multiple comparisons were applied.

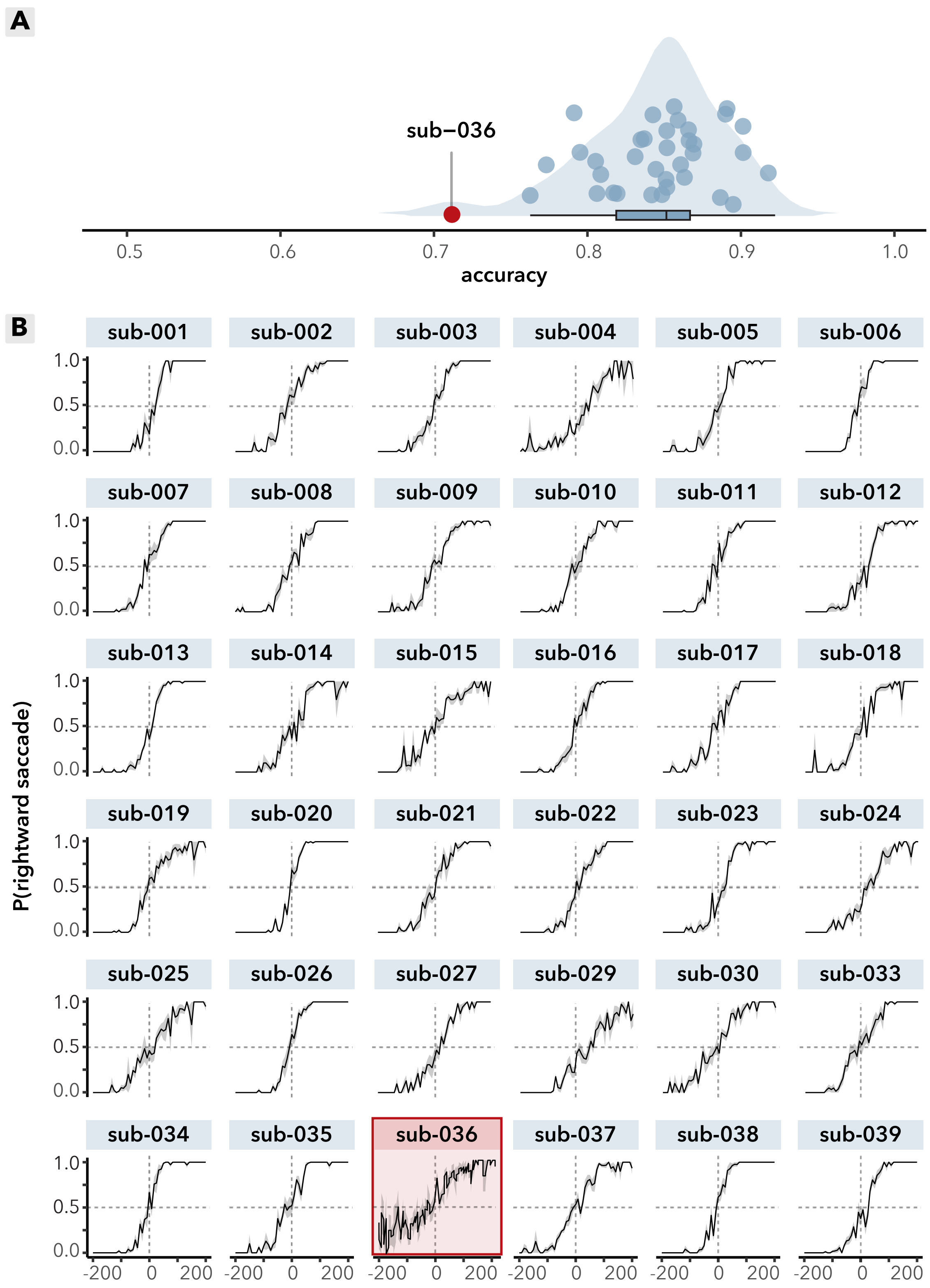
A
sub−036
0.5
0.6
0.7
0.8
0.9
1.0
accuracy
B
sub-001
sub-002
sub-003
sub-004
sub-005
sub-006
sub-007
sub-008
sub-009
sub-010
sub-011
sub-012
sub-013
sub-014
sub-015
sub-016
sub-017
sub-018
sub-019
sub-020
sub-021
sub-022
sub-023
sub-024
sub-025
sub-026
sub-027
sub-029
sub-030
sub-033
sub-034
sub-035
sub-036
sub-037
sub-038
sub-039
P(rightward saccade)
1.0
0.5
0.0
-200
0
200

Figure S4 | Participant accuracy and psychometric quality checks

(A) Distribution of overall accuracy across participants. The shaded curve is a kernel-density estimate; the horizontal boxplot shows the median (line), quartiles (box), and whiskers. Participants were flagged as outliers if they met any robust criterion: (i) Tukey IQR rule (accuracy < Q1 − 1.5·IQR or > Q3 + 1.5·IQR), (ii) |z| > 3 based on the sample mean/SD, and/or (iii) robust MAD-z > 3.5. The red marker highlights sub-036 that exceeded all three criteria and is flagged as an outlier. **(B)** Empirical psychometric curves plotting the probability of a rightward saccade against SOAs (ms; rightward-positive), collapsed across all conditions. Light grey ribbons indicate the S.E.M. across trials per SOA bin. Most participants show the expected monotonic increase from leftward to rightward delays (horizontal dashed line at 0.5 = chance, and vertical dashed line at 0 SOA). In contrast, sub-036 (red panel) exhibits non-monotone, noisy behavior with extended near-chance performance, consistent with non-compliance/unstable responding. Source data are provided as a Source Data file.

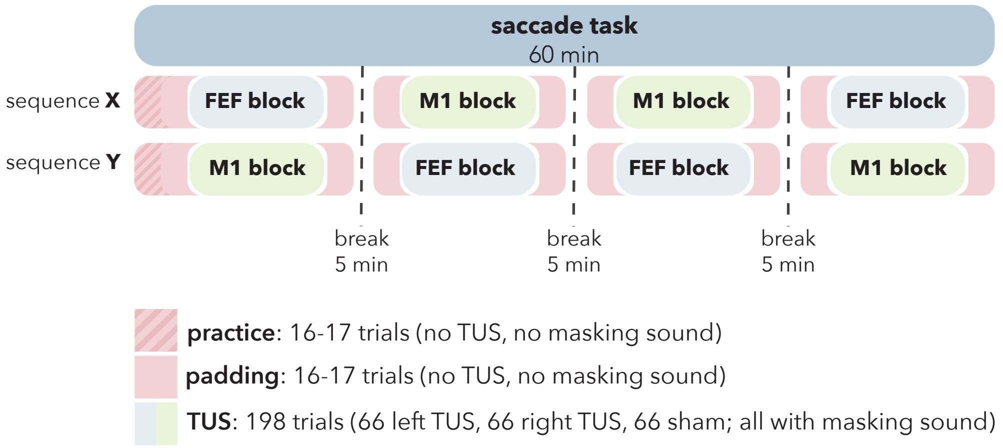

Figure S5 | Schematic overview of the saccade task duration and block sequences

The task consists of two randomized and counterbalanced block sequences: X (FEF, M1, M1, and then FEF) and Y (M1, FEF, FEF, and M1), with the order of the blocks varying across participants. Each block comprises 198 trials (66 left TUS, 66 right TUS, and 66 sham trials, all with masking sound), with additional padding of 16-17 trials before and after the block, where no TUS or masking sound is presented. At the start of each session, participants complete a short practice block for task familiarization. A 5-minute break is provided between blocks for participants to stretch and rest before transducers are recoupled. FEF stimulation is represented in blue and M1 stimulation in green.

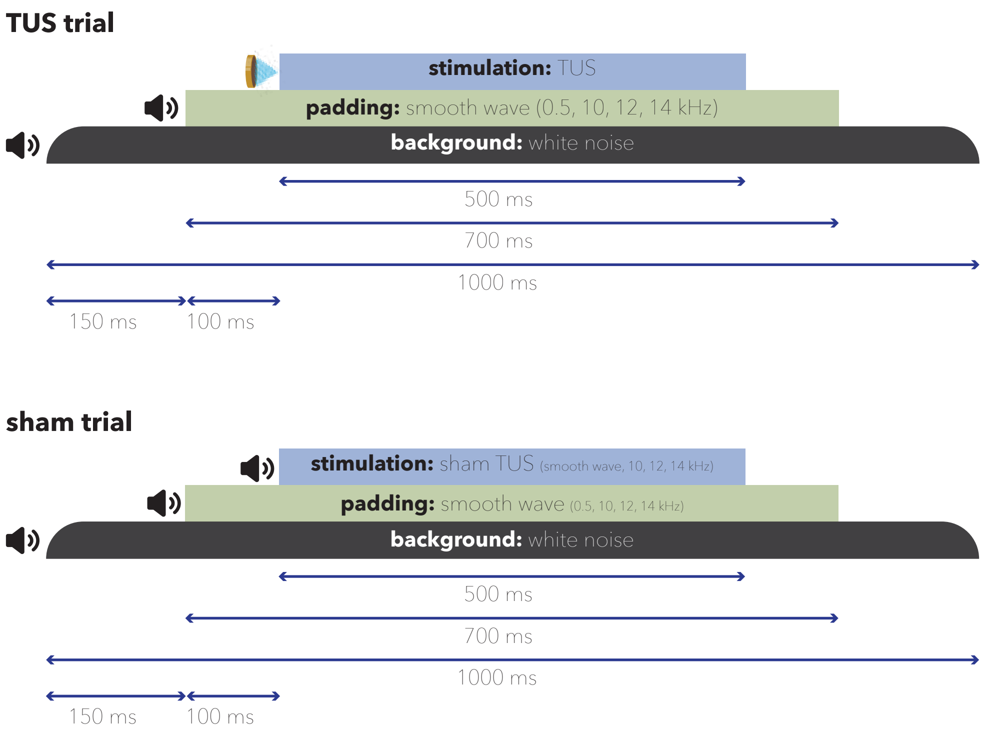

Figure S6 | Schematic overview of masking structure in TUS and sham trials

The trial begins with background white noise, delivered via bone-conducting headphones, lasting 1000 ms. 150 ms after the onset of the white noise, a smooth wave (padding sound) consisting of frequencies 0.5, 10, 12, and 14 kHz is delivered via bone-conducting headphones for 700 ms. For TUS trials (top), the 500 ms ultrasonic stimulation is then delivered 250 ms after the white noise onset (100 ms after the padding sound). For sham trials (bottom), the 500 ms of ultrasonic stimulation is replaced by a smooth wave of 10, 12, and 14 kHz, mimicking the TUS sound.

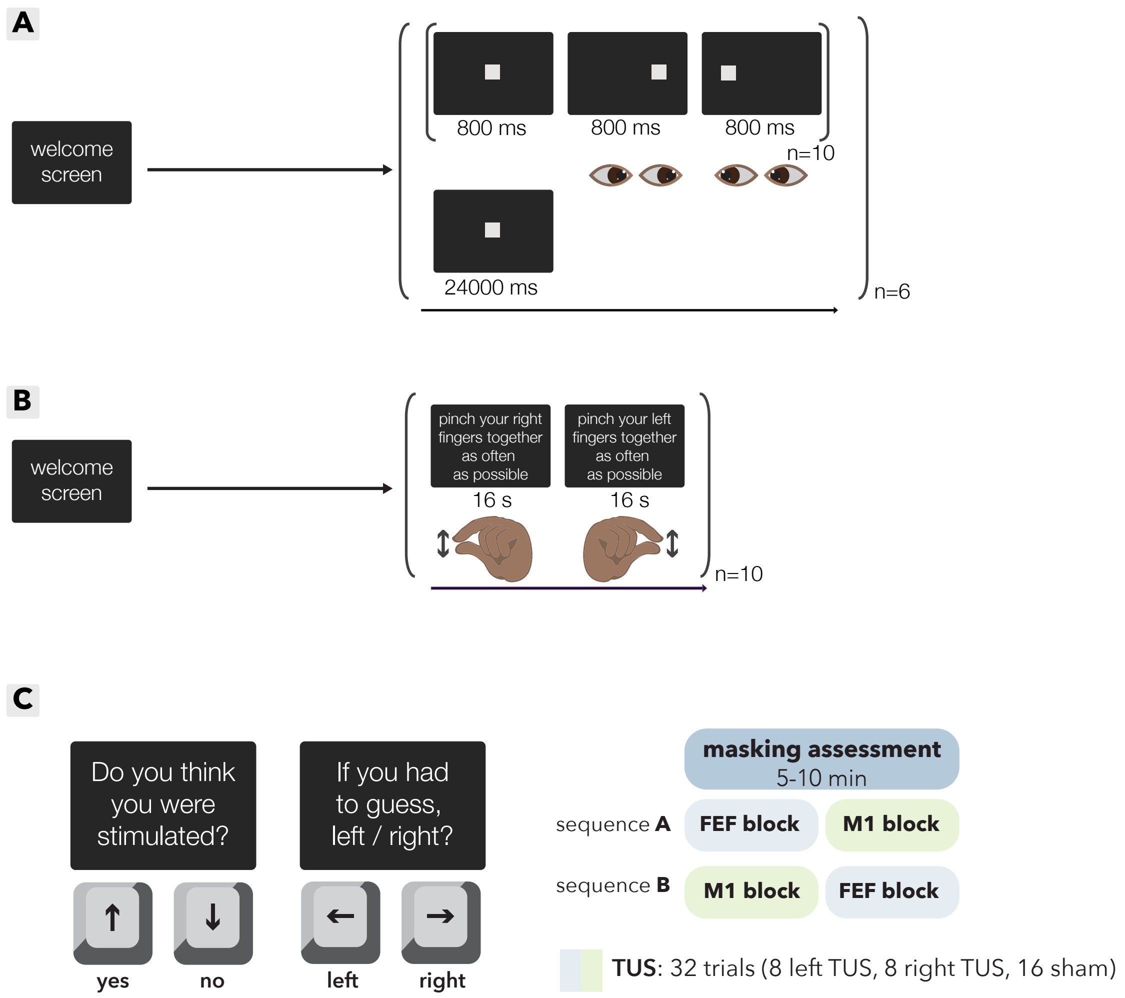

Figure S7 | Schematic overview of functional localizers and masking assessment

(A) FEF functional localizer. After a welcome screen, participants completed blocks of "follow the target" and "fixate on the target". In the 'follow the target' blocks, participants were presented with targets in a random order of left, center, and right positions. This sequence was randomized and repeated 10 times per block, with each target being shown for 800 ms. In the "fixation" block, the target was displayed in the center for 24 seconds. This sequence was repeated six times. The contrast was fixation versus follow the target. **(B)** M1 functional localizer. After a welcome screen, two blocks were presented, repeated 10 times. In each block, participants were instructed to either pinch their right index finger and thumb as often as possible, or their left index finger and thumb as often as possible for 16 seconds. The contrast was left versus right. **(C)** Masking assessment. Participants received TUS on the left/right FEF, left/right M1, and sham stimulation. For each trial, participants were first asked to indicate whether they thought they had received stimulation by pressing the up-arrow key for yes and the down-arrow key for no. Afterward, they were asked to guess whether the stimulation was on the left or right side by pressing the left-arrow key for left and the right-arrow key for right. The order of the conditions/trials was randomized within each block, with the overall sequence determined by the block sequence (right panel). Each block comprises of 8 left TUS, 8 right TUS and 16 sham trials.

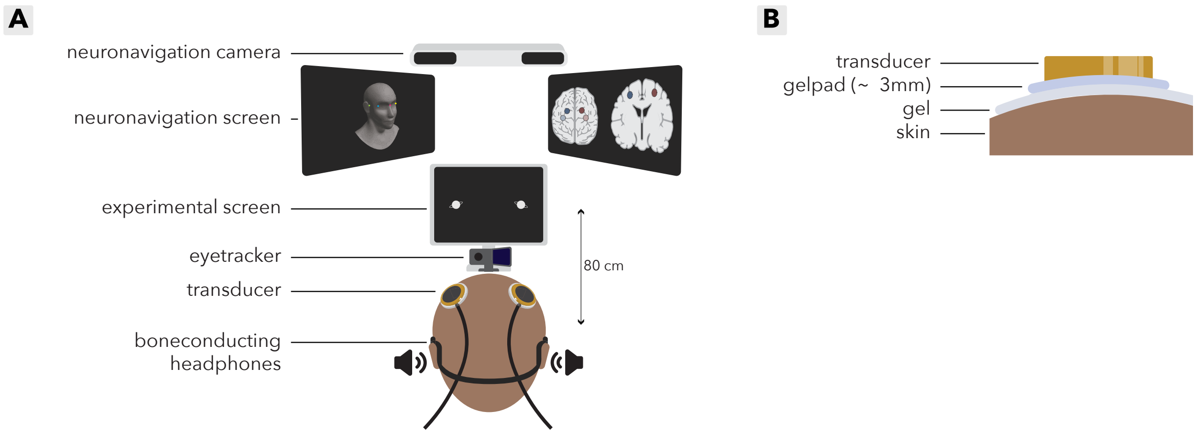

Figure S8 | Overview of the study setup

(A) Schematic overview of the TUS setup. Participants were seated 80 cm from the experimental task screen, with their head stabilized on a chinrest at the center of the screen. Transducers were positioned using a Velcro headcap and guided by neuronavigation for precise targeting (coordinates derived from FEF and M1 functional localizers and entered into Localite software). Bone-conducting headphones for masking were placed on participants, and an eye tracker was positioned below the screen to record eye movements. After neuronavigation, bone-conducting headphones were secured, and transducers were coupled to the participant's head while they remained still. Eye-tracking calibration was performed at the start of each block, prior to initiating the saccade task. **(B)** Schematic overview of coupling materials and layers. The participant's hair and scalp were carefully prepared with ultrasound gel to ensure full coverage of hair follicles and minimize air pockets. A thin (~3 millimeters) gel pad was placed on top of the ultrasound gel layer, allowing for visualization and removal of any remaining air bubbles. Another layer of ultrasound gel was applied to the transducer surface, ensuring no air bubbles were present. The transducer was then carefully positioned at the stimulation site, guided by neuronavigation for precise targeting.

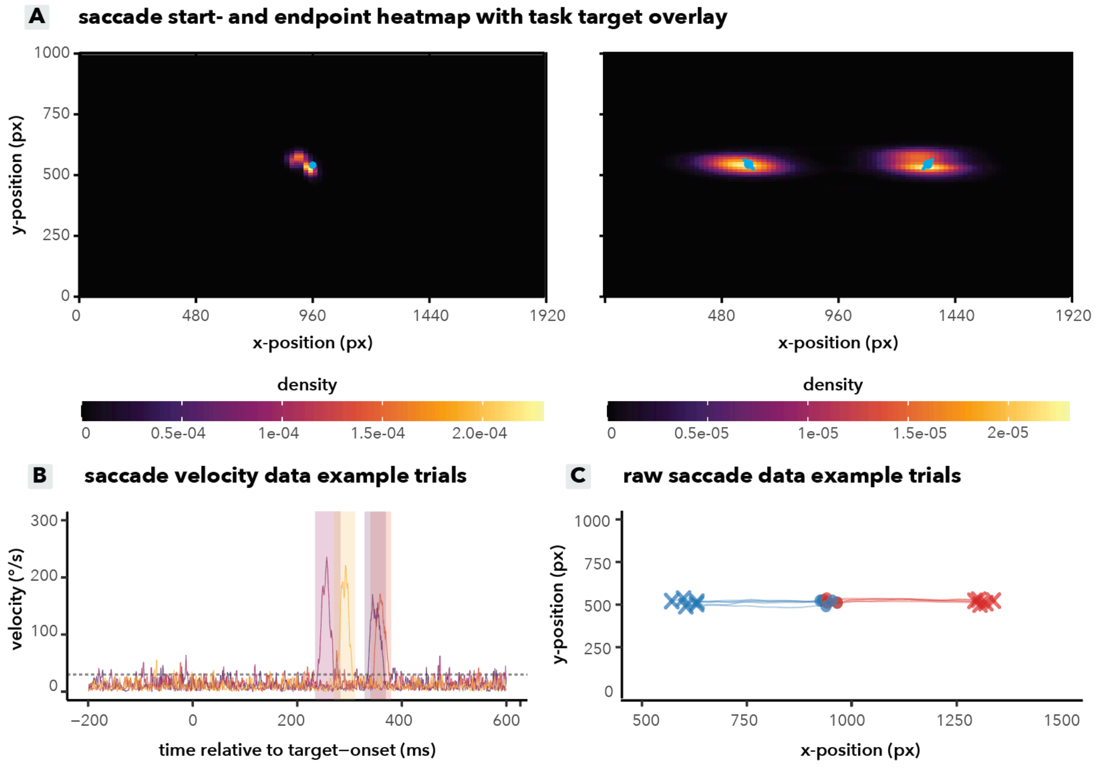

Figure S9 | Raw saccade examples and spatial distributions (example participant; *n* = 1)

(A) Heatmaps of saccade start positions (left) and endpoints (right) across all qualifying trials. Bright colors indicate higher density estimates. Blue overlays show the task visual targets: fixation at screen center (960 px) and left/right targets at ±10° from center (crosses). **(B)** Velocity–time traces (°/s) for the same example trials, aligned to target onset (t = 0 ms). Shaded rectangles (matching the line colors) indicate the detected saccade duration for each trial; peaks correspond to primary and, when present, corrective saccades. The horizontal dashed line denotes the velocity threshold used for saccade detection. **(C)** Overlaid raw examples of the first qualifying saccade after target onset from several trials. Filled circles mark saccade onset; × marks the endpoint. Thin lines show the raw gaze trace within each trial. Colors encode saccade direction (blue = leftward; red = rightward). Axes are gaze position in pixels. Source data are provided as a Source Data file.

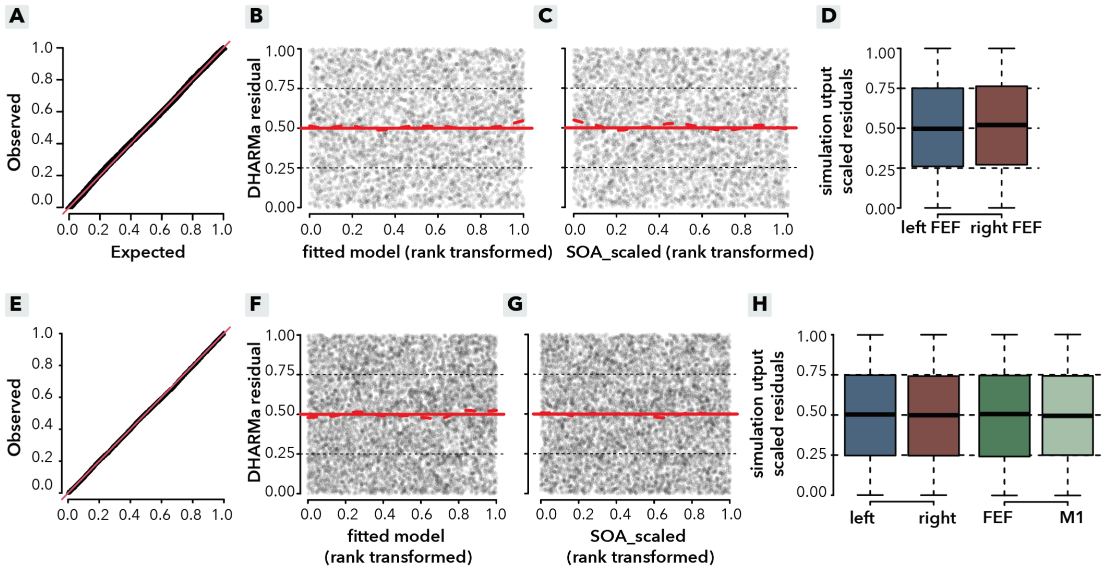

Figure S10 | GLMM diagnostics for two main statistical analyses

(A) FEF-choice domain model: DHARMa quantile–quantile (uniformity) plot of simulation-based residuals; Kolmogorov–Smirnov test: $p = 0.13389$ (n.s.); dispersion test: $p = 0.947$ (n.s.); outlier test: $p = 1$ (n.s.). **(B)** FEF-choice domain model: DHARMa residuals versus model predictions (both rank-transformed). The LOESS smooth (red) is approximately horizontal, indicating no global misfit or excess hetero(dis)persions beyond model expectation. **(C)** FEF-choice domain model: DHARMa residuals versus SOA_scaled (rank-transformed). The near-flat trend indicates no evidence of non-linearity in the logit with respect to SOA; a natural-spline alternative for SOA did not improve fit (LRT $\chi^2(2) = 0.52$, $p = 0.77$). **(D)** FEF-choice domain model: DHARMa residuals by stimSide (left FEF vs right FEF). Within-group deviation from uniformity: n.s.; Levene test for homogeneity of variance: n.s.; i.e., no heteroscedasticity across levels. **(E)** Side × Region model: DHARMa quantile–quantile (uniformity) plot of simulation-based residuals; Kolmogorov–Smirnov test: $p = 0.12922$ (n.s.); dispersion test: $p = 0.848$ (n.s.); outlier test: $p = 0.21016$ (n.s.). **(F)** Side × Region model: DHARMa residuals versus model predictions (both rank-transformed). The LOESS smooth (red) is approximately horizontal, indicating no global misfit or excess hetero(dis)persions beyond model expectation. **(G)** Side × Region model: DHARMa residuals versus SOA_scaled (rank-transformed). The near-flat trend indicates no evidence of non-linearity on the logit scale; a natural-spline alternative for SOA did not improve fit (LRT $\chi^2(2) = 0.626$, $p = 0.731$). **(H)** Side × Region model: DHARMa residuals by stimSide (left vs right). Within-group deviation from uniformity: n.s.; Levene test for homogeneity of variance: n.s. **(I)** Side × Region model: DHARMa residuals by stimRegion (FEF vs M1). Within-group deviation from uniformity: n.s.; Levene test for homogeneity of variance: n.s.

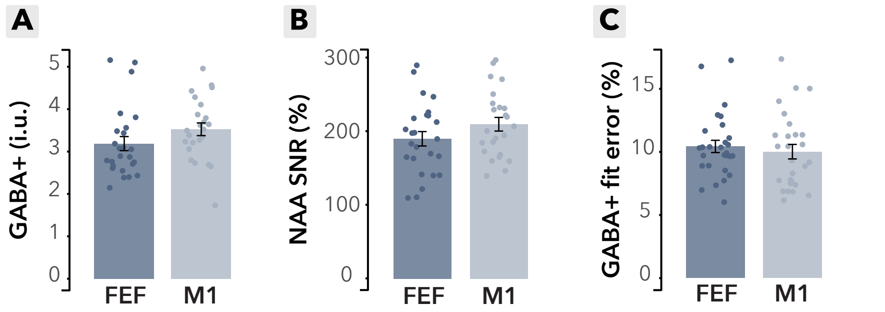

Figure S11 | MRS-derived measurements for the left FEF and left M1 Voxels

(A) Estimated GABA+ levels (in institutional units, i.u.) for each participant in the left FEF (blue) and left M1 (light blue) voxels, derived using Gannet. **(B)** N-Acetylaspartate (NAA) signal-to-noise ratio (SNR) (%) for each participant in the left FEF (blue) and left M1 (light blue) voxels. The NAA SNR reflects the strength and quality of the NAA peak relative to background noise and serves as an indicator of data quality. **(C)** GABA+ fit error (%) for each participant in the left FEF (blue) and left M1 (light blue) voxels. The fit error indicates the quality of the GABA+ estimation, with lower percentages reflecting better fits. Data file. **(A)**, **(B)**, **(C)** Each dot represents an individual participant, with bars showing the group mean and error bars indicating the standard error of the mean (SEM); n = 25. Source data are provided as a Source

Transducers and drive systems description

	model number, manufacturer	centre frequency	radius of curvature	aperature diameter	number elements	element distribution
transducer	NeuroFUS CTX250-009, Sonic Concepts	250 kHz	63.2 mm	45.5 mm	2	Annular array comprising a circular innter element and an annular outer element of equal area
matching integrated drive system	NeuroFUS TPO-105, Sonic Concepts					

	model number, manufacturer	centre frequency	radius of curvature	aperature diameter	number elements	element distribution
transducer	NeuroFUS CTX250-014, Sonic Concepts	250 kHz	63.2 mm	45.5 mm	2	Annular array comprising a circular innter element and an annular outer element of equal area
matching integrated drive system	NeuroFUS TPO-203, Sonic Concepts					

Drive system settings

operating frequency	output level setting	focal positioning setting
250 kHz	25 W/cm²(Isppa)	32.5 mm (FLHM)

Free field acoustic parameters

	model number, manufacturer	spatial-peak pressure amplitude	axial position spatial-peak pressure	position of centre of axial -3dB pressure	axial -3dB width	axial -6dB width
transducer	NeuroFUS CTX250-009, Sonic Concepts	0.9 MPa	30 mm	33 mm	25 mm	37 mm
transducer	NeuroFUS CTX250-014, Sonic Concepts	0.9 MPa	31 mm	34 mm	23 mm	34 mm

Pulse timing parameters

	duration	ramp duration	ramp shape	repitition interal / frequency
pulse	2 ms	1000 μs	tukey	2 ms / 500 Hz
pulse train	500 ms			

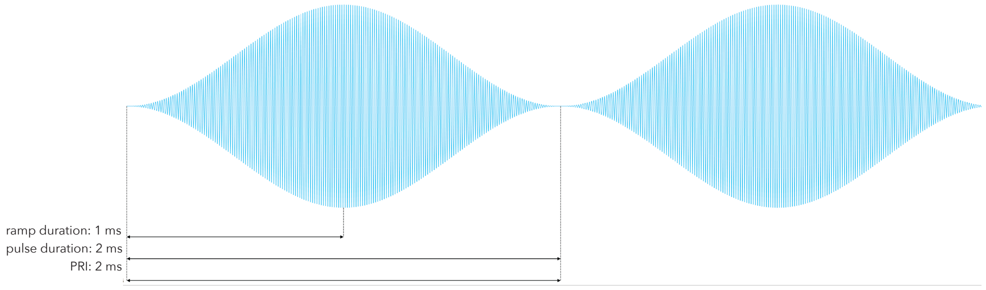

Intensity parameters

spatial-peak pulse-average intensity	spatial-peak time-average intensities	acoustic impedance
9.37W/cm²	9.37 W/cm²	1.5x10⁶ Rayls

Figure S12 | Transcranial ultrasonic stimulation standardized reporting details

Tables S1-S9: Additional analyses and statistical outcomes

This section provides a summary of key findings from supplementary statistical analyses:

Table S1 | Additional analyses and statistical outcomes examining the effect of condition and delay on choice behavior. The model was tested across three different choice domains (25%-75%, 20%-80%, and 15%-85%) to assess the robustness of the effects. Bold cells indicate column and row labels, while shaded blue cells highlight the main comparison outcomes. Statistical significance was determined using two-sided logistic mixed effects regressions. No multiple comparisons were applied.

**Model:**	$choice \sim condition_{(\text{left FEF, right FEF})} + SOA + (condition_{(\text{left FEF, right FEF})} + SOA \mid sub)$														
**Data:**	choice domain 25% - 75%					choice domain 20% - 80%					choice domain 15% - 85%				
	χ^2	**Df**	p	b	**95%-CI**	χ^2	**Df**	p	b	**95%-CI**	χ^2	**Df**	p	b	**95%-CI**
intercept	10	1	0.8	0.14	0.1, 0.2	12	1	<0.001	0.1	0.1, 0.2	13	1	<0.001	0.14	0.1, 0.2
LFEF – RFEF	10	1	0.001	-0.25	-0.4, -0.1	10	1	0.002	-0.2	-0.4, -0.1	8	1	0.005	-0.22	-0.4, -0.1
SOA	185	1	<0.001	1.62	1.4, 1.9	347	1	<0.001	2.2	2.0, 2.5	490	1	<0.001	2.82	2.6, 3.1
**Model:**	$choice \sim condition_{(\text{left M1, right M1})} + SOA + (condition_{(\text{left M1, right M1})} + SOA \mid sub)$														
**Data:**	choice domain 25% - 75%					choice domain 20% - 80%					choice domain 15% - 85%				
	χ^2	**Df**	p	b	**95%-CI**	χ^2	**Df**	p	b	**95%-CI**	χ^2	**Df**	p	b	**95%-CI**
intercept	0	1	0.9	0.0	-0.1, 0.1	0	1	0.6	0.0	-0.1, 0.1	3	1	0.09	0.07	-0.0, 0.1
LM1 – RM1	2	1	0.17	-0.1	-0.2, 0.0	3	1	0.10	-0.1	-0.2, 0.0	4	1	0.044	-0.14	-0.3, -0.0
SOA	187	1	<0.001	1.64	1.4, 1.9	359	1	<0.001	2.3	2.0, 2.5	415	1	<0.001	2.98	2.7, 3.3

Table S2 | Additional analyses and statistical outcomes examining the effect of condition and delay on choice behavior, excluding trials where the target delay was 0 seconds. The model was tested across three different choice domains (25%-75%, 20%-80%, and 15%-85%) to assess the robustness of the effects. Bold cells indicate column and row labels, while shaded blue cells highlight the main comparison outcomes. Statistical significance was determined using two-sided logistic mixed effects regressions. No multiple comparisons were applied.

**Model:**	$choice \sim condition_{(\text{left FEF, right FEF})} + SOA + (condition_{(\text{left FEF, right FEF})} + SOA \mid sub)$														
**Data:**	choice domain 25% - 75%					choice domain 20% - 80%					choice domain 15% - 85%				
	χ^2	**Df**	p	b	**95%-CI**	χ^2	**Df**	p	b	**95%-CI**	χ^2	**Df**	p	b	**95%-CI**
intercept	7	1	0.007	0.12	0.0, 0.2	9	1	0.002	0.13	0.0, 0.2	11	1	<0.001	0.13	0.1, 0.2
LFEF – RFEF	8	1	0.004	-0.24	-0.4, -0.1	8	1	0.005	-0.22	-0.4, -0.1	6	1	0.012	-0.20	-0.4, -0.0
SOA	175	1	<0.001	1.60	1.4, 1.8	339	1	<0.001	2.20	2.0 – 2.4	481	1	<0.001	2.82	2.6, 3.1
**Model:**	$choice \sim condition_{(\text{left M1, right M1})} + SOA + (condition_{(\text{left M1, right M1})} + SOA \mid sub)$														
**Data:**	choice domain 25% - 75%					choice domain 20% - 80%					choice domain 15% - 85%				
	χ^2	**Df**	p	b	**95%-CI**	χ^2	**Df**	p	b	**95%-CI**	χ^2	**Df**	p	b	**95%-CI**
intercept	1	1	0.4	-0.04	-0.1, 0.1	0	1	0.8	-0.01	-0.1, 0.1	1	1	0.3	0.04	-0.0, 0.1
LM1 – RM1	0	1	0.8	-0.02	-0.1, 0.1	1	1	0.5	-0.05	-0.2, 0.1	2	1	0.2	-0.09	-0.2, 0.0
SOA	175	1	<0.001	1.63	1.4, 1.9	347	1	<0.001	2.28	2.0, 2.5	390	1	<0.001	2.98	2.7, 3.3

Table S3 | Additional analyses and statistical outcomes examining the effect of stimulation side, stimulation region and delay on choice behavior, including an interaction between side and region. The model was tested across three different choice domains (25%-75%, 20%-80%, and 15%-85%) to assess the robustness of the effects. Bold cells indicate column

and row labels, while shaded blue cells highlight the main comparison outcomes. Statistical significance was determined using two-sided logistic mixed effects regressions. No multiple comparisons were applied.

**Model:**	*choice ~ side$_{(left, right)}$ x region$_{(FEF, M1)}$ + SOA + (side$_{(left, right)}$ x region$_{(FEF, M1)}$ + SOA | sub)*														
**Data:**	choice domain 25% - 75%					choice domain 20% - 80%					choice domain 15% - 85%				
	**χ^2**	**Df**	***p***	***b***	**95%-CI**	**χ^2**	**Df**	***p***	***b***	**95%-CI**	**χ^2**	**Df**	***p***	***b***	**95%-CI**
intercept	9	1	0.003	0.14	0.0, 0.2	8	1	0.004	0.15	0.0, 0.2	6	1	0.011	0.14	0.0, 0.2
side	12	1	<0.001	-0.26	-0.4, -0.1	12	1	<0.001	-0.25	-0.4, -0.1	9	1	0.003	-0.22	-0.4, -0.1
region	5	1	0.032	-0.14	-0.3, -0.0	14	1	0.039	-0.12	-0.2, -0.0	2	1	0.14	-0.08	-0.2, 0.0
SOA	310	1	<0.001	1.63	1.4, 1.8	480	1	<0.001	2.27	2.1, 2.5	557	1	<0.001	2.93	2.7, 3.2
side x region	3	1	0.09	0.16	-0.0, 0.4	3	1	0.11	0.14	-0.0, 0.3	1	1	0.03	0.09	-0.1, 0.3

Table S4 | Additional analyses and statistical outcomes examining the effect of stimulation side, stimulation region and delay on choice behavior, including an interaction between side and region, excluding trials where target delay was 0 seconds. The model was tested across three different choice domains (25%-75%, 20%-80%, and 15%-85%) to assess the robustness of the effects. Bold cells indicate column and row labels, while shaded blue cells highlight the main comparison outcomes. Statistical significance was determined using two-sided logistic mixed effects regressions. No multiple comparisons were applied.

**Model:**	*choice ~ side$_{(left, right)}$ x region$_{(FEF, M1)}$ + SOA + (side$_{(left, right)}$ x region$_{(FEF, M1)}$ + SOA | sub)*														
**Data:**	choice domain 25% - 75%					choice domain 20% - 80%					choice domain 15% - 85%				
	**χ^2**	**Df**	***p***	***b***	**95%-CI**	**χ^2**	**Df**	***p***	***b***	**95%-CI**	**χ^2**	**Df**	***p***	***b***	**95%-CI**
intercept	7	1	0.009	0.13	0.0, 0.2	7	1	0.009	0.13	0.0, 0.2	6	1	0.018	0.13	0.0, 0.2
side	9	1	0.003	-0.25	-0.4, -0.1	9	1	0.002	-0.24	-0.4, -0.1	7	1	0.008	-0.21	-0.4, -0.1
region	6	1	0.012	-0.18	-0.3, -0.0	5	1	0.020	-0.14	-0.3, -0.0	3	1	0.09	-0.10	-0.2, 0.0
SOA	288	1	<0.001	1.61	1.4, 1.8	468	1	<0.001	2.25	2.0, 2.5	534	1	<0.001	2.91	2.7, 3.2
side x region	5	1	0.025	0.23	0.0, 0.4	4	1	0.038	0.19	0.0, 0.4	2	1	0.15	0.13	-0.0, 0.3

Table S5 | Additional analyses and statistical outcomes examining the effect of stimulation side, stimulation region, presence of zero-delay trials and delay on choice behavior, including an interaction between side and region. The model was tested across three different choice domains (25%-75%, 20%-80%, and 15%-85%) to assess the robustness of the effects. Bold cells indicate column and row labels, while shaded blue cells highlight the main comparison outcomes. Statistical significance was determined using two-sided logistic mixed effects regressions. No multiple comparisons were applied.

**Model:**	*choice ~ side$_{(left, right)}$ x region$_{(FEF, M1)}$ x delay0 + SOA + (side$_{(left, right)}$ x region$_{(FEF, M1)}$ + SOA | sub)*														
**Data:**	choice domain 25% - 75%					choice domain 20% - 80%					choice domain 15% - 85%				
	**χ^2**	**Df**	***p***	***b***	**95%-CI**	**χ^2**	**Df**	***p***	***b***	**95%-CI**	**χ^2**	**Df**	***p***	***b***	**95%-CI**
intercept	7	1	0.008	0.13	0.0, 0.2	10	1	0.002	0.13	0.0, 0.2	5	1	0.020	0.13	0.0, 0.2
side	10	1	0.001	-0.25	-0.4, -0.1	9	1	0.003	-0.24	-0.4, -0.1	8	1	0.006	-0.21	-0.4, -0.1
region	6	1	0.014	-0.17	-0.3, -0.0	4	1	0.040	-0.14	-0.3, -0.0	3	1	0.09	-0.10	-0.2, 0.0
delay 0	0	1	0.5	0.08	-0.2, 0.4	0	1	0.5	0.8	-0.2, 0.3	0	1	0.5	0.08	-0.2, 0.3
SOA	309	1	<0.001	1.63	1.4, 1.8	476	1	<0.001	2.26	2.1, 2.5	554	1	<0.001	2.92	2.7, 3.2
side x region	5	1	0.033	0.22	0.0, 0.4	3	1	0.066	0.19	-0.0, 0.4	2	1	0.15	0.13	-0.0, 0.3
side x del 0	0	1	0.74	-0.06	-0.4, 0.3	0	1	0.6	-0.09	-0.4, 0.3	0	1	0.6	-0.10	-0.5, 0.3
region x del 0	2	1	0.16	0.26	-0.1, 0.6	2	1	0.2	0.23	-0.1, 0.6	1	1	0.3	0.18	-0.2, 0.5
side x region x delay 0	4	1	0.058	-0.50	-1.0, 0.0	3	1	0.09	-0.45	-1.0, 0.1	2	1	0.14	-0.38	-0.9, 0.1

Table S6 | Additional analyses and statistical outcomes examining the interaction between condition and baseline GABA+ levels in the FEF on choice behavior. The model was tested across three different choice domains (25%-75%, 20%-80%, and 15%-85%) to assess the robustness of the effects. Bold cells indicate column and row labels, while shaded blue cells highlight the main comparison outcomes. Statistical significance was determined using two-sided logistic mixed effects regressions. No multiple comparisons were applied.

**Model:**	*choice ~ condition$_{(left\ FEF,\ sham)}$ x FEF_GABA + SOA + (condition$_{(left\ FEF,\ sham)}$ + SOA | sub)*														
**Data:**	choice domain 25% - 75%					choice domain 20% - 80%					choice domain 15% - 85%				
	**χ^2**	**Df**	***p***	***b***	**95%-CI**	**χ^2**	**Df**	***p***	***b***	**95%-CI**	**χ^2**	**Df**	***p***	***b***	**95%-CI**
condition	6	1	0.011	0.64	0.1, 1.2	9	1	0.003	0.66	0.2, 1.2	9	1	0.003	0.65	0.2, 1.1
FEF GABA	2	1	0.12	0.12	0.0, 0.2	1	1	0.3	0.10	-0.0, 0.2	2	1	0.14	0.16	0.0, 0.3
SOA	269	1	<0.001	1.76	1.5, 2.0	433	1	<0.001	2.23	2.0, 2.4	509	1	<0.001	2.88	2.6, 3.1
condition x FEF GABA	3	1	0.070	-0.15	-0.3, 0.0	4	1	0.050	-0.15	-0.3, -0.0	4	1	0.037	-0.15	-0.3, -0.0
sham x FEF GABA	5	1	0.019	0.12	0.0, 0.2	5	1	0.022	0.10	0.0, 0.2	4	1	0.050	0.15	0.0, 0.3
LFEF x FEF GABA	0	1	0.6	-0.03	-0.2, 0.1	0	1	0.5	-0.05	-0.2, 0.1	0	1	0.9	0.01	-0.2, 0.2

Table S7 | Additional analyses and statistical outcomes examining the interaction between condition and baseline GABA+ levels in the FEF on choice behavior, excluding trials where target delay was 0 seconds. The model was tested across three different choice domains (25%-75%, 20%-80%, and 15%-85%) to assess the robustness of the effects. Bold cells indicate column and row labels, while shaded blue cells highlight the main comparison outcomes. Statistical significance was determined using two-sided logistic mixed effects regressions. No multiple comparisons were applied.

**Model:**	*choice ~ condition$_{(left\ FEF,\ sham)}$ x FEF_GABA + SOA + (condition$_{(left\ FEF,\ sham)}$ + SOA | sub)*														
**Data:**	choice domain 25% - 75%					choice domain 20% - 80%					choice domain 15% - 85%				
	**χ^2**	**Df**	***p***	***b***	**95%-CI**	**χ^2**	**Df**	***p***	***b***	**95%-CI**	**χ^2**	**Df**	***p***	***b***	**95%-CI**
condition	5	1	0.021	0.84	0.3, 1.4	8	1	0.005	0.84	0.3,1.4	8	1	0.004	0.79	0.3, 1.3
FEF GABA	2	1	0.13	0.14	0.0, 0.2	1	1	0.4	0.11	0.0, 0.2	2	1	0.19	0.16	0.0, 0.03
SOA	274	1	<0.001	1.75	1.5, 2.0	432	1	<0.001	2.21	2.0, 2.4	546	1	<0.001	2.87	2.6, 3.1
condition x FEF GABA	6	1	0.017	-0.21	-0.4, -0.0	6	1	0.011	-0.21	-0.4, -0.0	6	1	0.011	-0.19	-0.3, -0.0
sham x FEF GABA	7	1	0.008	0.14	0.0, 0.2	4	1	0.041	0.11	0.0, 0.2	4	1	0.035	0.16	0.0, 0.3
LFEF x FEF GABA	1	1	0.3	-0.08	-0.2, 0.1	2	1	0.18	-0.09	-0.2, 0.0	0	1	0.7	-0.03	-0.2, 0.1

Table S8 | Additional analyses and statistical outcomes examining the interaction between condition and baseline GABA+ levels in the M1 on choice behavior. The model was tested across three different choice domains (25%-75%, 20%-80%, and 15%-85%) to assess the robustness of the effects. Bold cells indicate column and row labels, while shaded blue cells highlight the main comparison outcomes. Statistical significance was determined using two-sided logistic mixed effects regressions. No multiple comparisons were applied.

**Model:**	$choice \sim condition_{(left\ M1,\ sham)}\ x\ M1\_GABA + SOA + (condition_{(left\ M1,\ sham)} + SOA \mid sub)$														
**Data:**	choice domain 25% - 75%					choice domain 20% - 80%					choice domain 15% - 85%				
	**χ^2**	**Df**	***p***	***b***	**95%-CI**	**χ^2**	**Df**	***p***	***b***	**95%-CI**	**χ^2**	**Df**	***p***	***b***	**95%-CI**
condition	0	1	1.0	-0.24	-0.9, 0.4	0	1	0.7	-0.22	-0.9, 0.5	1	1	0.4	-0.01	-0.6, 0.6
M1 GABA	0	1	0.1	-0.03	-0.2, 0.1	1	1	0.3	-0.06	-0.2, 0.0	1	1	0.4	-0.09	-0.3, 0.1
SOA	191	1	<0.001	1.72	1.5, 2.0	264	1	<0.001	2.24	2.0, 2.5	369	1	<0.001	2.97	2.7, 3.3
condition x M1 GABA	1	1	0.5	0.07	-0.1, 0.3	1	1	0.5	0.07	-0.1, 0.3	0	1	0.8	0.02	-0.1, 0.2
sham x M1 GABA	0	1	0.5	-0.04	-0.2, 0.1	1	1	0.3	-0.06	-0.2, 0.1	1	1	0.4	-0.08	-0.3, 0.1
LM1 x M1 GABA	0	1	0.7	0.03	-0.1, 0.2	0	1	0.9	-0.01	-0.2, 0.2	1	1	0.4	-0.07	-0.2, 0.1

Table S9 | Additional analyses and statistical outcomes examining the interaction between condition and baseline GABA+ levels in the M1 on choice behavior, excluding trials where target delay was 0 seconds. The model was tested across three different choice domains (25%-75%, 20%-80%, and 15%-85%) to assess the robustness of the effects. Bold cells indicate column and row labels, while shaded blue cells highlight the main comparison outcomes. Statistical significance was determined using two-sided logistic mixed effects regressions. No multiple comparisons were applied.

**Model:**	$choice \sim condition_{(left\ M1,\ sham)}\ x\ M1\_GABA + SOA + (condition_{(left\ M1,\ sham)} + SOA \mid sub)$														
**Data:**	choice domain 25% - 75%					choice domain 20% - 80%					choice domain 15% - 85%				
	**χ^2**	**Df**	***p***	***b***	**95%-CI**	**χ^2**	**Df**	***p***	***b***	**95%-CI**	**χ^2**	**Df**	***p***	***b***	**95%-CI**
condition	0	1	0.6	-0.24	-1.0, 0.5	0	1	1.0	0.66	0.2, 1.2	0	1	0.7	0.01	-0.6, 0.6
M1 GABA	0	1	0.8	-0.01	-0.1, 0.1	0	1	0.8	0.10	-0.0, 0.2	1	1	0.5	-0.06	-0.2, 0.1
SOA	185	1	<0.001	1.71	1.5, 2.0	251	1	<0.001	2.23	2.0, 2.4	364	1	<0.001	2.96	2.7, 3.3
condition x M1 GABA	0	1	0.6	0.06	-0.1, 0.3	0	1	0.5	-0.15	-0.3, -0.0	0	1	1.0	0.01	-0.2, 0.2
sham x M1 GABA	0	1	0.9	-0.01	-0.1, 0.1	0	1	0.6	-0.03	-0.2, 0.1	0	1	0.5	-0.06	-0.2, 0.1
LM1 x M1 GABA	0	1	0.5	0.06	-0.1, 0.2	0	1	0.9	0.01	-0.2, 0.2	0	1	0.6	-0.05	-0.2, 0.1
