# Supplementary material for: Rapid modulation of choice behavior by ultrasound on the human frontal eye fields
Source: Nat Commun. 2026 Feb 20;17:2966. doi: 10.1038/s41467-026-69854-7 (PMC13035901; doi:10.1038/s41467-026-69854-7)
Supplement: Supplementary file 2 — Reporting Summary [file 41467_2026_69854_MOESM2_ESM.pdf]

nature portfolio

Corresponding author(s): Soha Farboud

Last updated by author(s): 29/08/2025

Reporting Summary

Nature Portfolio wishes to improve the reproducibility of the work that we publish. This form provides structure for consistency and transparency in reporting. For further information on Nature Portfolio policies, see our Editorial Policies and the Editorial Policy Checklist.

Statistics

For all statistical analyses, confirm that the following items are present in the figure legend, table legend, main text, or Methods section.

n/a	Confirmed	
☐	☒	The exact sample size (*n*) for each experimental group/condition, given as a discrete number and unit of measurement
☐	☒	A statement on whether measurements were taken from distinct samples or whether the same sample was measured repeatedly
☐	☒	The statistical test(s) used AND whether they are one- or two-sided<br>*Only common tests should be described solely by name; describe more complex techniques in the Methods section.*
☐	☒	A description of all covariates tested
☐	☒	A description of any assumptions or corrections, such as tests of normality and adjustment for multiple comparisons
☐	☒	A full description of the statistical parameters including central tendency (e.g. means) or other basic estimates (e.g. regression coefficient) AND variation (e.g. standard deviation) or associated estimates of uncertainty (e.g. confidence intervals)
☐	☒	For null hypothesis testing, the test statistic (e.g. *F*, *t*, *r*) with confidence intervals, effect sizes, degrees of freedom and *P* value noted<br>*Give P values as exact values whenever suitable.*
☒	☐	For Bayesian analysis, information on the choice of priors and Markov chain Monte Carlo settings
☒	☐	For hierarchical and complex designs, identification of the appropriate level for tests and full reporting of outcomes
☐	☒	Estimates of effect sizes (e.g. Cohen's *d*, Pearson's *r*), indicating how they were calculated

Our web collection on statistics for biologists contains articles on many of the points above.

Software and code

Policy information about availability of computer code

Data collection	Stimulus presentation and TUS triggering were implemented in PsychoPy v2021.2.3 with custom Python scripts. We provided separate task scripts for (i) the main saccade task, (ii) the masking assessment, and (iii) the fMRI localizers (FEF/M1). Trial timing and TUS triggers were logged on each trial. Eye movements were recorded with an EyeLink 1000 Plus; MRI was acquired on a Siemens 3T Magnetom Skyra with a 32-channel head coil. Localite neuronavigation registered individual localizer peaks to anatomy and guided transducer placement. All task code (main task, masking, FEF/M1 localizers) is publicly available as indicated in the manuscript's Data/Code availability statement: https://doi.org/10.34973/drtg-kq58
Data analysis	fMRI localizers were preprocessed/analyzed in SPM12 (MATLAB R2023a). Spectroscopy was processed with Gannet 3.1.4 (tissue fractions from SPM12). Behavioral statistics were run in RStudio (v4), using the packages lme4 (v1.1-37), emmeans (v1.11.1), and DHARMa (v.0.4.7). All scripts for preprocessing and statistical analysis are available at the same project repository referenced in the manuscript's Data/Code availability statement: https://doi.org/10.34973/drtg-kq58

For manuscripts utilizing custom algorithms or software that are central to the research but not yet described in published literature, software must be made available to editors and reviewers. We strongly encourage code deposition in a community repository (e.g. GitHub). See the Nature Portfolio guidelines for submitting code & software for further information.

Data

Policy information about availability of data

All manuscripts must include a data availability statement. This statement should provide the following information, where applicable:

- Accession codes, unique identifiers, or web links for publicly available datasets
- A description of any restrictions on data availability
- For clinical datasets or third party data, please ensure that the statement adheres to our policy

All data generated and analyzed during this study are included in the manuscript and supporting files. Upon publication the behavioral data, group-level data and both task and analysis codes are available via the Radboud University Data Sharing Platform via https://doi.org/10.34973/drtg-kq58

Research involving human participants, their data, or biological material

Policy information about studies with human participants or human data. See also policy information about sex, gender (identity/presentation), and sexual orientation and race, ethnicity and racism.

Reporting on sex and gender	We recorded sex (biological attribute) as self-reported female or male for sample description only; gender identity was not collected. The final behavioral sample comprised 15 females and 20 males, all right-handed (mean age 24.1 ± 3.2 y). Sex were not part of the study hypotheses, and the work was not powered for sex- based comparisons; accordingly, no sex stratified analyses were performed. Given the within-subject design targeting acute, lateralized FEF effects, and the absence of strong a-priori predictions for sex differences in these specific metrics, including sex as factors would not be interpretable at this sample size. Aggregate sex counts are reported above; de-identified participant-level metadata (including sex) are available as indicated in the manuscript's Data availability statement.
Reporting on race, ethnicity, or other socially relevant groupings	Not applicable.
Population characteristics	We studied healthy, right-handed young adults with a final behavioral sample of N = 35 (mean age 24.1 ± 3.2 years; 15 female / 20 male; age range 20–32) with no history of neurological/psychiatric disorders, serious head trauma, epilepsy/seizure, or implanted metal; participants were screened for MRI/TUS safety and asked to abstain from recreational drugs (48 h) and >4 alcoholic units (24 h) before testing. The study used a within-subject design (no parallel groups); session order and stimulation blocks were counterbalanced, and time-of-day was held constant per participant.
Recruitment	Healthy, right-handed adults were recruited primarily via Radboud University channels and additionally via local word-of-mouth; the sample was not limited to RU students. After medical and MRI/TUS safety screening (no neurological/psychiatric history, no metal implants, no recent alcohol/drug use), 39 participants were enrolled; 35 completed the protocol and entered the main analyses (Mage ≈ 24; 15 female/20 male). The study used a double-blind, within-subject design with counterbalanced block sequences and brief practice to ensure task comprehension. As a convenience sample of young adults, some self-selection and age/education biases are possible; hand dominance was restricted to right-handers to reduce variance in hemispheric lateralization.
Ethics oversight	All participants gave written informed consent in accordance with the Declaration of Helsinki. Procedures were approved by the regional ethics committee Commissie Mensgebonden Onderzoek (CMO) Oost-Nederland (protocol CMO2022-15953).

Note that full information on the approval of the study protocol must also be provided in the manuscript.

Field-specific reporting

Please select the one below that is the best fit for your research. If you are not sure, read the appropriate sections before making your selection.

☒ Life sciences ☐ Behavioural & social sciences ☐ Ecological, evolutionary & environmental sciences

For a reference copy of the document with all sections, see nature.com/documents/nr-reporting-summary-flat.pdf

Life sciences study design

All studies must disclose on these points even when the disclosure is negative.

Sample size	We targeted a within-subject design with N = 35 complete datesets for the behavioral analyses (MRS subset smaller due to standard QC). The target N was set a priori based on feasibility and power considerations for the preregistered primary contrasts (left-vs-right FEF; Side × Region [FEF vs M1]). To accommodate drop-out/technical loss (eye-tracking/MRS), we enrolled 39 participants; 35 completed the protocol and entered the main analyses.
Data exclusions	Participant-level. Of 39 enrolled, 3 were excluded due to eye-tracking instability/technical failure (frequent signal loss/recalibration; one did not complete the task). These cases were identified during data collection and replaced, which is why recruitment continued beyond the target until 39 were enrolled. 1 additional participant was excluded as an accuracy outlier across the main task (pre-specified robust criterion; indicative of non-compliance/non-monotone behavior rather than momentary uncertainty). The final behavioral sample was N = 35.

	Trial-level (behavior). Practice trials were not analyzed. For each trial we selected the first qualifying saccade after target onset; trials were discarded if (i) start position was outside the fixation window, (ii) the endpoint did not land in exactly one lateral ROI, (iii) a blink overlapped the saccade, (iv) tracking was missing/saturated, or (v) no qualifying saccade occurred in the response window. Confirmatory GLMMs operated on a participant-specific "choice domain" (≈25–75% choice proportion) to isolate low-evidence decisions; robustness windows (20–80%, 15–85%) are reported in the Supplement. SOA = 0 trials were not entered into confirmatory models (descriptive only).
Replication	Reproducibility was addressed by (i) a preregistered analysis plan with confirmatory primary contrasts, (ii) internal replication across hemispheres (left vs right FEF) and against an active spatial control (M1) plus sham, (iii) robustness checks across alternative choice-domain windows (20–80%, 15–85%) and complementary psychometric models (slope/bias), and (iv) full code/data availability to enable re-analysis. All primary, preregistered effects (contralateral bias during FEF-TUS; Side × Region interaction) were observed as predicted; exploratory after-effect analyses were underpowered and are reported with CIs.
Randomization	This was a within-subject study; no parallel groups were formed. To control order effects, stimulation site/order was counterbalanced (blocks targeting FEF and M1 were presented in opposite orders across participants). Within each block, trial types (left-TUS, right-TUS, sham) were presented in a pseudorandom sequence with equal counts and constraints on immediate repetitions. SOA values were randomized on each trial with stratified oversampling of short SOAs. In the final TUS session, the masking assessment started with either FEF or M1 (order randomized and counterbalanced across participants); within each masking block, left-TUS, right-TUS, and sham trials were randomized. For the localizers, trials were randomized as well: in the M1 localizer (fMRI), left- vs right-finger presses were randomized; in the FEF localizer, left- vs right-saccade directions were randomized.
Blinding	Blinding differed by level:<br><br>Within-block (trial level): double-blind. Participants and the task operator were blind to left-TUS, right-TUS, or sham on each trial; waveforms were triggered from coded scripts, and setup (placement, timing, auditory mask) was identical across trial types. This level corresponds to our primary comparison (e.g., left vs right FEF).<br><br>Block/site level (FEF vs M1): single-blind. Participants were blind to the stimulated site, but the operator necessarily knew the site to position the transducer. Block order was counterbalanced and the site information was not revealed to participants. The masking assessment followed the same pattern: site single-blind, trial type double-blind.<br><br>Analysis blinding. Post-hoc blinding checks indicated that although participants could sometimes distinguish TUS vs sham, they could not reliably identify stimulated side/site, and detection performance did not account for the FEF-specific behavioral effects.

Reporting for specific materials, systems and methods

We require information from authors about some types of materials, experimental systems and methods used in many studies. Here, indicate whether each material, system or method listed is relevant to your study. If you are not sure if a list item applies to your research, read the appropriate section before selecting a response.

Materials & experimental systems

n/a	Involved in the study
☒	☐ Antibodies
☒	☐ Eukaryotic cell lines
☒	☐ Palaeontology and archaeology
☒	☐ Animals and other organisms
☒	☐ Clinical data
☒	☐ Dual use research of concern
☒	☐ Plants

Methods

n/a	Involved in the study
☒	☐ ChIP-seq
☒	☐ Flow cytometry
☐	☒ MRI-based neuroimaging

Plants

Seed stocks	Not applicable.
Novel plant genotypes	Not applicable.
Authentication	Not applicable.

Magnetic resonance imaging

Experimental design

Design type	Task-based fMRI with block designs for both functional localizers. The FEF localizer alternated blocks of visually guided saccades and central fixation; the M1 localizer alternated blocks of left- and right-hand finger pinching.
Design specifications	For the FEF localizer, participants followed a target that stepped randomly among left/center/right positions; each target was shown for 800 ms and 30 steps formed a 24-s "saccade" block, alternating with 24-s fixation blocks; this sequence was repeated six times. The GLM modeled saccade-vs-fixation blocks convolved with the canonical HRF.<br><br>For the M1 localizer, participants alternated 16-s blocks of repetitive pinching with the left vs right hand, six blocks per hand (12 blocks total); GLMs contrasted left- vs right-hand movement to localize each M1.<br><br>All localizer EPI used multiband MB4 (TR = 995 ms, TE = 32.8 ms, voxel 2.5 mm isotropic).
Behavioral performance measures	These runs served purely as functional localizers; no behavioral performance metrics were recorded. Compliance was ensured by instructions and verified via the expected single-subject BOLD contrasts—saccade > fixation to define FEF and contralateral M1 activation (left-hand > right-hand and vice versa) to define M1.

Acquisition

Imaging type(s)	Structural (T1-weighted, T2-weighted, and UTE), functional BOLD fMRI (localizers), and single-voxel proton MRS (MEGA-PRESS/PRESS).
Field strength	3 T Magnetom Skyra (Siemens) with a 32-channel head coil.
Sequence & imaging parameters	T1w (structural): TR = 2700 ms, TE = 3.69 ms, flip angle = 9°, voxel = 0.9×0.9×0.9 mm, FOV = 230 mm.<br><br>T2w (structural): TR = 3200 ms, TE = 408 ms, variable flip-angle mode, voxel = 0.9×0.9×0.9 mm, FOV = 230 mm.<br><br>UTE (structural): TR = 3.32 ms, TE = 0.07 ms, flip angle = 2°, voxel = 0.8×0.8×0.8 mm, FOV = 294 mm.<br><br>fMRI localizers (MB4 EPI): TR = 995 ms, TE = 32.8 ms, flip angle = 60°, voxel = 2.5×2.5×2.5 mm, FOV = 210×210×130 mm (axial).<br><br>MRS (left FEF/M1): MEGA-PRESS for GABA+ (TR = 2000 ms, TE = 68 ms, 2.0×2.0×2.0 cm voxel, VAPOR water suppression; 128 averages + 16 water-unsuppressed); PRESS for Glx (TR = 20000 ms, TE = 35 ms, 2.0×2.0×2.0 cm voxel, VAPOR; 64 averages).
Area of acquisition	Whole-brain coverage for structural scans (T1w/T2w/UTE). fMRI localizers used an axial FOV of 210×210×130 mm providing coverage of frontal and parietal cortices to include FEF and M1; peak voxels within the resulting activation clusters were used to define individual stimulation targets. MRS voxels (2×2×2 cm) were placed in left FEF and left M1 based on anatomical landmarks and the T1w image.

Diffusion MRI ☐ Used ☒ Not used

Preprocessing

Preprocessing software	Functional localizer data were pre-processed and analyzed in SPM12 (MATLAB R2023a); results were visualized with MRIcroGL. The first five volumes of each run were discarded to allow steady-state magnetization; IMA files were converted and all runs were visually inspected for artefacts. Realignment and reslicing were performed, images were coregistered to each participant's T1-weighted anatomy (single-subject analyses), and smoothed with a 6 mm FWHM Gaussian kernel.<br><br>GABA+ spectra were processed with Gannet 3.1.4 (frequency/phase correction by spectral registration, line broadening; tissue fractions from SPM12; relaxation/tissue correction), with independent visual QC and exclusion if >50% averages required removal.
Normalization	For group-level maps, functional data were spatially normalized to MNI standard space using SPM's normalization after coregistration to the participant's T1-weighted image. Single-subject coordinates were retained in native space for neuronavigation.
Normalization template	MNI (Montreal Neurological Institute) standard space.
Noise and artifact removal	Motion was corrected via SPM realignment (parameters inspected); data were smoothed (6 mm FWHM) and the first five volumes were removed to avoid pre-steady-state artefacts. No additional structured noise regressors are reported in the manuscript.
Volume censoring	No volume "scrubbing"/censoring was applied beyond the removal of the first five volumes; analyses proceeded after realignment and visual QC.

Statistical modeling & inference

Model type and settings	Mass-univariate first-level GLMs in SPM12. Block regressors (saccade vs fixation for the FEF localizer; left-hand vs right-hand pinching for the M1 localizer) were convolved with the canonical HRF (SPM defaults), included a high-pass filter (128 s) and AR(1) temporal autocorrelation. Six head-motion parameters were entered as nuisance covariates. Maps were computed per participant (single-subject), as the localizers served to define individual ROIs/targets for TUS.
Effect(s) tested	FEF localizer: saccade > fixation (and the reverse for QC).<br>M1 localizer: left-hand > right-hand and right-hand > left-hand to identify contralateral M1.<br>Peaks within the expected anatomy were used to define individual stimulation targets; no whole-brain group inference was performed from these contrasts.

Specify type of analysis: ☐ Whole brain ☒ ROI-based ☐ Both

Anatomical location(s)	ndividual ROIs were defined with single-subject functional localizers at the intake scan. For FEF, we modeled saccades > fixation and selected the peak voxel within the significant cluster constrained to the medial FEF at the junction of the superior precentral sulcus and superior frontal sulcus (an anatomically informed choice given its role in higher-order oculomotor control). For M1, we modeled right-finger > left-finger and left-finger > right-finger pinching blocks and took the local peak within the hand area of the precentral "hand knob." Preprocessing/first-level GLMs were done in SPM12, smoothed 6 mm FWHM, with FWE-corrected cluster inference; peaks were visually verified in FSLeyes and then imported into Localite for neuronavigation during TUS.

Statistic type for inference<br>(See Eklund et al. 2016)	Voxel-wise t-contrasts at the single-subject level. For localization purposes, peaks were selected from thresholded maps ($p < 0.001$ uncorrected, $k \geq 10$ voxels) within the a-priori anatomical territory (FEF or M1). No cluster-wise correction was used because the maps were not used for group-level inference.
Correction	Because the localizer contrasts were used only to define subject-specific ROIs rather than to test group hypotheses, no multiple-comparison correction at the group level was required. Single-subject thresholding (voxel-wise $p < 0.001$ uncorrected, small extent threshold) ensured robust peak identification within the a-priori regions.

Models & analysis

n/a	Involved in the study
☒	☐ Functional and/or effective connectivity
☒	☐ Graph analysis
☒	☐ Multivariate modeling or predictive analysis
